# Serum Resistance of *Mycoplasma agalactiae* Strains and Mutants Bearing Different Lipoprotein Profiles

**DOI:** 10.3390/pathogens11091036

**Published:** 2022-09-13

**Authors:** Katja Sommer, Saskia Kowald, Rohini Chopra-Dewasthaly

**Affiliations:** Department of Pathobiology, Institute of Microbiology, University of Veterinary Medicine Vienna, 1210 Vienna, Austria

**Keywords:** mycoplasmas, serum resistance, antigenic phase variation, complement resistance, immune evasion

## Abstract

In order to spread systemically, resistance against complement and other factors present in serum is an important trait in pathogenic bacteria. The variable proteins of *Mycoplasma agalactiae* (Vpmas) have been shown to affect differential adhesion, invasion and immune evasion, and undergo high-frequency phase-variation in expression. However, nothing is known about their involvement in *M. agalactiae’*s serum susceptibility. To evaluate this, the PG2 strain, the GM139 strain and the six Vpma phase-locked mutants (PLMs, PLMU to PLMZ) were tested for their ability to survive in the presence of non-sensitized and sensitized sheep serum, as well as guinea pig complement. Additionally, the reactivity of the sensitized sheep serum was analysed on the strains via western blotting. Overall data demonstrate PG2 strain to be more susceptible to sheep serum compared to the GM139 strain bearing a different Vpma profile. Significant differences were also observed between the different PLMs, with PLMU and PLMX showing the highest serum susceptibility in serum, while the other PLMs expressing longer Vpma proteins were more resistant. The results are in good correlation with previous studies where shorter lipoprotein variants contributed to a higher susceptibility to complement. Since none of the tested strains and PLMs were susceptible to non-sensitized sheep serum, antibodies seem to play an important role in serum killing.

## 1. Introduction

Mycoplasmas are one of the smallest, self-replicating bacteria. Due to degenerative evolution, they lack not only different biosynthetic pathways but also a cell wall [1]. In spite of their minimal genomes of 580 to 1840 kilobases [2], large multigene families of variable lipoproteins have been retained in many different species, such as *Mycoplasma pulmonis, M. bovis* and *M. agalactiae*, suggesting their importance for pathogenesis [3,4,5]. The different factors in pathogenicity have yet to be further elucidated, however, these variable lipoproteins can aid in the avoidance of the host immune system by undergoing high-frequency phase variation [6]. Additionally, they might play a role in serum resistance, which is an important aspect of systemic spread during infection.

*M. agalactiae* exhibits high-frequency antigenic variation of the family of surface lipoproteins called Vpmas (Variable Proteins of *M. agalactiae*) or Avg (Agalactiae Variable Gene) proteins [7,8]. These have been shown to be involved in adhesion, cell invasion [9] and immune evasion [10]. Each *vpma* gene consists of unique and shared amino acid sequences, with the 5′ untranslated and N-terminal regions being especially conserved [11]. These *vpma* genes are site-specifically rearranged by the Xer1 recombinase, which subsequently leads to differential expression of the antigens. The Vpma size can be varied through DNA slippage and domains shuffled via DNA recombination and gene duplication [3]. Under in vivo selection pressure in the immunocompetent host and in the presence of corresponding Vpma-specific antibodies in vitro, however, Xer1 independent Vpma switching has been observed via the formation of chimeras, duplications and deletions. This proves the role of Vpmas in immune evasion [10,12]. In the type strain PG2 of *M. agalactiae*, six different *vpma* genes are present (*vpma*U-Z). Their translated products range in size from about 22–23 kDa (VpmaU and VpmaX) to about 33–35 kDa (VpmaV, VpmaW, VpmaY, VpmaZ) based on the gene sequences [6]. While the *vpma* composition is similar in most other *M. agalactiae* strains, rare duplication events can happen [13]. However, almost nothing was known about the Vpma composition and expression in GM139 strain until a recent report where it has been shown to express only VpmaV without evident phase variation in western and colony blots. The underlying *vpma* sequence however has not yet been investigated [14].

Through targeted disruption of the Xer1 recombinase in the PG2 strain, Vpma phase-locked mutants (PLMs) were generated, so that only one of the respective Vpmas is constitutively expressed without further switching [6]. In vitro studies with PLMs have demonstrated, that PLMV, followed by PLMW is highly adhesive, while PLMU was least adhesive. The invasion of cells by the PLMs seems to be closely correlated to their adhesion rate. This highlights the decreased fitness of PLMU during host colonization [9,12]. Wild type strains capable of Vpma phase variation, for example PG2, have been suggested to be more invasive and therefore better at spreading systemically in vivo than PLMU and PLMY in a sheep trial [15]. The dominance of PLMY over PLMU at local infection sites during experimental co-challenge studies indicates a better in vivo fitness for PLMY [12].

Since the ability to evade the host immune system and to successfully survive in the blood stream are closely correlated, different serum resistance mechanisms prove to be essential for causing systemic infections [16]. In *M. pulmonis*, the variable surface antigens (Vsa) are involved in the steric shielding of the mycoplasmal surface. Mycoplasmas bearing Vsa proteins with an increased length of 40–60 tandem repeats have an enhanced resistance to both lysis through the membrane attack complex (MAC) [17], and to phagocytosis [18], while cells with repeats of five or less are more susceptible. Other bacteria like *Haemophilus influenzae, Staphylococcus aureus* or *Mycoplasma hypopneumoniae* have developed surface proteins to inhibit C3b opsonization or MAC assembly [19,20,21]. For *M. agalactiae*, serum resistance has only been described in connection to the variable expression of a β-(1 → 6)-glucopyranose structure on the cell surface, which greatly reduced survival [22]. Therefore, the Vpma profile of different *M. agalactiae* strains and mutants might also impact serum resistance and differential pathogenicity. The current study is the first attempt to evaluate the same using different strains and mutants bearing different Vpma profiles.

## 2. Results

### 2.1. Bactericidal Assays with Sheep Sera and Guinea Pig Serum Complement Reveal Significant Differences in Susceptibilty and Relative Survival

#### 2.1.1. Sensitized Sheep Serum

*M. agalactiae* strains and PLMs displayed differential survival after treatment with sheep sera obtained 17 days after experimental infection with *M. agalactiae* (D+17 p.i.).

**Figure 1:**
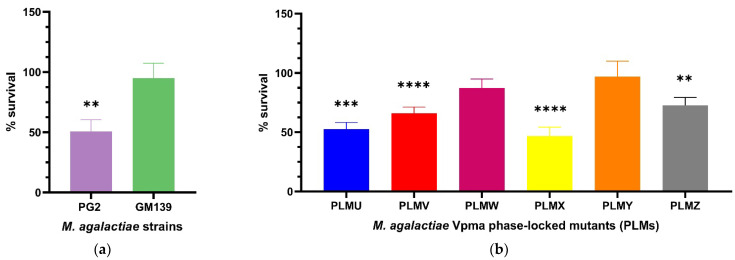
Statistically significant serum killing of individual *M. agalactiae* wild-type strains (**a**) and six different Phase-Locked Mutants (PLMs) (**b**) in presence of *M. agalactiae*-sensitized sheep serum (obtained on Day 17 post infection) compared to the corresponding heat-inactivated serum control. *M. agalactiae* field strain GM139, Phase-Locked Mutant W (PLMW) and Phase-Locked Mutant Y (PLMY) did not show statistically significant differences in the CFU/mL of their respective cultures treated with heat-inactivated control and active serum. In contrast, type strain PG2 (** *p* < 0.01), Phase-Locked Mutant V (PLMV) and Phase-Locked Mutant X (PLMX) (**** *p* < 0.0001), Phase-Locked Mutant U (PLMU) (*** *p* < 0.001), and Phase-Locked Mutant Z (PLMZ) (** *p* < 0.01) demonstrated significant differences in the same, and hence exhibited significant serum killing. Comparative statistical differences between the means of survival percentages for the PLMs are additionally illustrated in Supplementary Figure S2.

Percentage survival was calculated as shown in Section 4.4. The use of the T_0_ sample to take into account the starting CFU/mL counts at the beginning of the experiments did not lead to any prominent change in the results as also observed in a previous study [23], and was therefore only performed in the first experiments to confirm this. The GM139 strain did not show significant differences in CFU/mL counts for the complement and heat-inactivated *M. agalactiae* sensitized serum (D+17) treated cultures and exhibited a percentage survival of 95%. On the other hand, almost half of the PG2 population died and displayed significant killing in presence of this serum (** *p* < 0.01) as depicted in Figure 1a. The difference in the percentage survival of GM139 and PG2 was even far more significant (**** *p* < 0.0001) as shown in Supplementary Figure S1.

PLMs expressing single stable Vpma proteins demonstrated significant differences in their serum susceptibility (Figure 1b). PLMU, PLMV, PLMX and PLMZ showed significant susceptibility whereas almost no killing was observed for PLMW and PLMY. For the different PLMs, the survival averages varied, for instance, PLMU demonstrated a survival of 52.5%, PLMV 65.8%, PLMW 87.3%, PLMX 46.7%, PLMY 97.1%, and PLMZ 72.6%. As visible in Figure 1b, PLMU and PLMX were the most susceptible to serum and PLMY was the least affected. Statistically significant serum killing is calculated based on the differences in the CFU/mL of the culture treated with heat-inactivated serum as compared to the corresponding active serum. For instance, as shown in Figure 1b, although PLMV shows better survival (65.8%) than PLMU (52.5%), it exhibits statistically more significant serum killing (**** *p* < 0.0001) as the difference in the CFU/mL of its culture treated with heat-inactivated serum and the corresponding active serum was starker compared to PLMU (*** *p* < 0.001). Mean survival percentages also differed significantly between almost all PLMs, except for PLMU vs. PLMX, PLMV vs. PLMZ and PLMW vs. PLMY, as depicted in Supplementary Figure S2.

#### 2.1.2. Comparison of Non-Sensitized and Sensitized Sheep Serum

**Figure 2:**
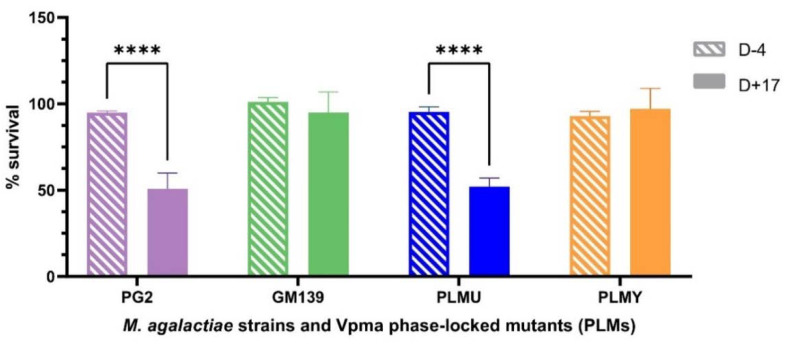
Comparative survival differences of *M. agalactiae* type strain PG2, field strain GM139, Phase-Locked Mutant U (PLMU) and Phase Locked Mutant Y (PLMY) treated with non-sensitized (D-4) and *M. agalactiae*-sensitized (D+17) sheep serum obtained on Day 4 before, and Day 17 post infection, respectively, and the corresponding heat-inactivated controls. Type strain PG2 and PLMU showed significantly higher serum killing (**** *p* < 0.0001) in the sensitized serum (D+17) compared to the non-sensitized (D-4) serum.

Unlike the sensitized sheep serum that was obtained after bleeding the animals at the end of the experimental infection after euthanizing them, only a very limited amount of blood could be withdrawn prior to infection to ensure their wellbeing for ongoing infection trials. Hence, due to the limited availability of the sheep serum obtained four days before inoculation (D-4), it was tested only on PG2, GM139, PLMU and PLMY. Although none of these strains and mutants showed susceptibility to this serum, PG2 and PLMU showed stark survival differences (**** *p* < 0.0001) between the D+17 sensitized and D-4 non-sensitized serum (Figure 2), further highlighting their higher susceptibility.

#### 2.1.3. Guinea Pig Serum Complement

**Figure 3:**
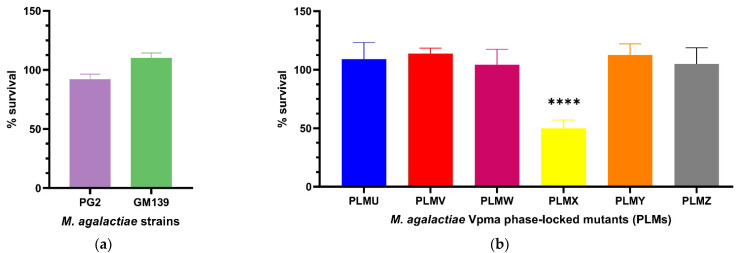
Statistically significant serum killing of individual *M. agalactiae* strains PG2 and GM139 (**a**) and six different Phase-Locked Mutants (PLMs) (**b**) in presence of guinea pig serum complement when compared to the corresponding heat-inactivated serum control. Neither the two wild-type strains, nor the PLMs showed any significant serum killing except for Phase-Locked Mutant X (PLMX), which showed a significantly lower CFU/mL in active guinea pig serum complement compared to the heat-inactivated control (**** *p* < 0.0001). Comparative survival differences between the PLMs are shown in Supplementary Figure S4.

In order to elaborate on the exact serum or complement factors affecting mycoplasma susceptibility, a more standardized commercial serum was thought to be ideal, especially for reproducibility, and also to negate the role of *M. agalactiae*-specific antibodies in serum killing. After treatment with guinea pig serum complement, the CFU/mL of neither PG2 nor GM139 differed significantly compared to the corresponding heat inactivated serum controls. PG2 had a mean survival of 92% and GM139 had a mean survival of 110% (see Figure 3a), and this difference between the percentage survival of these two strains was significant (**** *p* < 0.0001, Supplementary Figure S3). Similarly, all tested PLMs were resistant to active guinea pig serum complement as they did not show significant changes in the CFU/mL when their respective cultures were treated with active versus heat-inactivated complement, except for PLMX, which displayed a survival of only 50% (Figure 3b). Mean survival values also differed significantly between PLMX and the other respective PLMs as visible in Supplementary Figure S4.

### 2.2. Western Blot with Sheep Serum D+17

**Figure 4:**
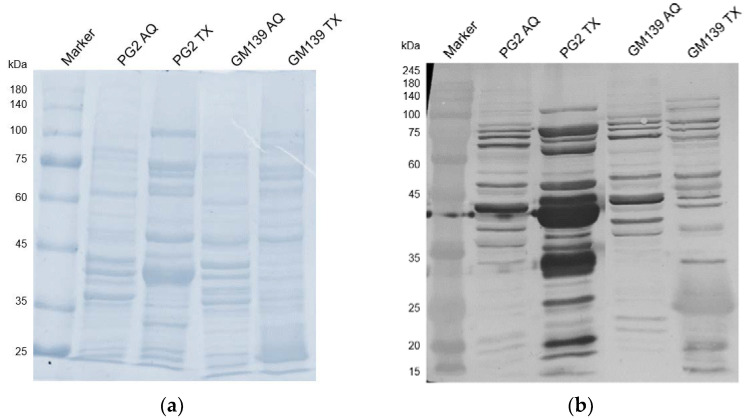
SDS-PAGE (**a**) and western blot analysis (**b**) of triton (TX) and aqueous (AQ) phases of *M. agalactiae* strains PG2 and GM139 treated with *M. agalactiae* PG2-sensitized sheep serum obtained on the 17th day of infection (D+17).

To analyze the comparative reactivity of the sensitized sheep serum to GM139 and PG2 strains, triton and aqueous phase extracts of both strains were probed with the D+17 sheep serum to check the antibody reactivity with the respective protein extracts. While only a few bands are visible in the aqueous phases (cytoplasmic proteins), as expected the membrane proteins (in the triton phase) are recognized extensively as visible in Figure 4. This is especially true for the PG2 strain, where several bands, including the ones at around 43 kDa and 34 kDa, are extremely strong. These likely correspond with the abundantly expressed Vpma proteins that usually run at similar positions in SDS-PAGE, which is a little higher than their actual molecular weight [6]. For the GM139 strain, most proteins are comparatively less strongly recognized except for one band at around 25 kDa.

## 3. Discussion

To spread systemically and to colonize tissues distant from the site of infection, resistance to complement and evasion of the cellular and humoral immune system of the blood is necessary [16,24].

Neither PG2 nor GM139 were susceptible to pre-immune serum and guinea pig serum complement, which means that complement and non-sensitized immune factors likely do not suffice to elicit a significant bactericidal response. In the PG2-sensitized serum, however, PG2 showed only 50% survival, meaning that antibodies or other factors present only in the sensitized serum likely play an essential role in the serum-killing of the PG2 strain. For *M. bovis*, complement killing was strongest in the presence of antibodies against surface proteins which activate the classical complement pathway and promote phagocytosis and C3b deposition [25]. Similarly, in the case of *E. coli*, only sensitized serum led to killing [26]. As can be seen in the western blot treated with D+17 sheep serum, there is extensive reactivity for the PG2-bands at expected Vpma sizes and also for GM139 bands, though fewer and fainter than for PG2. This reveals, that *M. agalactiae*-specific antibodies are present in the sensitized sheep serum and highlights the immunogenicity of Vpmas and similar surface proteins. Apparently, there is a stronger and more frequent binding to proteins in the PG2-triton-phase. This could be due to a higher immunogenicity of the PG2 proteins when compared to GM139, or simply because the serum was obtained from a PG2-sensitized sheep and therefore more PG2-specific antibodies are present.

PLMU and PLMX were the most susceptible in sensitized serum and PLMX was the only mutant sensitive to guinea pig serum complement, which fits the hypothesis that isogenic variants expressing smaller Vpmas are more affected by the serum killing, as also proven for *M. pulmonis* expressing shorter variants of Vsa lipoproteins [17]. PLMU however was not susceptible to pre-immune serum, which contrasts with this theory. A possible cause for this could be that the serum killing might be largely dependent on opsonization with antibodies or activation of complement via the classical pathway. Depending on the current Vpma expression during the experimental inoculation with PG2, different proportions of antibodies against the Vpmas could be present in the D+17 serum, which could explain the differential susceptibility. All the PLMs expressing longer Vpmas (PLMV, PLMW, PLMY, PLMZ) were less sensitive to sheep serum. This could be due to a better shielding of the important antigenic epitopes from antibodies and/or complement by longer Vpmas as compared to the shorter ones or due to charge effects. Moreover, other factors affecting complement susceptibility and the activation of the complement cascade might be present on the mycoplasmal membrane. Due to the length differences of the Vpmas, although small, the access to these could be altered between the PLMs [17,27]. PLMY had a higher survival rate, both in the sensitized and the non-sensitized serum, which is in line with previous studies where PLMY was dominant over PLMU in co-challenge infection studies in sheep via the intramammary and conjunctival routes, and in vitro adhesion assays [9,12,28]. Therefore, PLMY seems to be better equipped to withstand the host immune factors, including those in host serum, as compared to PLMU.

For the variable surface antigens (Vsa) of *M. pulmonis*, a strain closely related to *M. agalactiae*, strains producing Vsa proteins with more and longer tandem repeats are less susceptible to complement. While the complement cascade was activated both in strains with shorter and longer tandem repeats, the serum susceptibility differed, which indicates an inhibition of the MAC-formation conferring the complement resistance. Longer Vsa proteins prevented access of larger molecules to the mycoplasmal membrane. However, the size variation for Vsa is between 200 kDa to 32 kDa, a much bigger range as compared to the range of Vpmas (from 35 kDa to 22 kDa theoretical size based on gene sequences). Therefore, size variation alone might not be playing such an important role in differential PLM survival [17,18].

In previous experiments [23], both GM139 and PG2 were found susceptible to the sensitized serum obtained from a sheep 17 days after experimental infection with PLMZ, whereas GM139 was observed to be more resistant than PG2 to the non-sensitized serum from the same sheep. PG2 exhibited similar survival after treatment with both the non-sensitized and the sensitized serum at around 40% while PLMU was resistant to both sera. PLMY displayed about 55% survival for sensitized serum. Even though the percentage survival for the PG2 strain was rather similar to the current results, in this previous study the pre-immune serum caused stronger killing. This might be due to differences in the immune status of the animals, leading to dissimilar immune effectors, for examples immune cells or serum protein concentration [29].

An additional variable in serum resistance assays is the heat-inactivation of serum, because complement is not the only protein component inactivated in this process. Other antimicrobial factors like phospholipases, lysozyme and amidases, which aid in bacterial killing are inactivated as well. This means that several other elements, besides complement, affect killing even in unsensitized serum [30]. Therefore, specific complement inhibitors are necessary to determine the role of complement-killing in unsensitized serum. Additionally, using C1s inhibitors would allow to further study whether the observed increased killing in sensitized serum is solely due to antibody-activated complement pathway or possibly due to other killing pathways also enhanced by antibodies, for instance phagocytosis.

## 4. Materials and Methods

### 4.1. Bacterial Strains and Culture Conditions

*M. agalactiae* type strain PG2 (Madrid, Spain) [31], strain GM139 (CA, USA) [32], which had previously demonstrated sole expression of VpmaV, as well as the different Vpma phase-locked mutants of *M. agalactiae* (PLMs) [9] were tested in serum bactericidal assays. The cultures were grown at 37 °C in SP-4 broth for 24 h or SP-4 agar for 3–5 days supplemented with penicillin (500 U/mL) for PG2 and GM139 and with tetracycline (2 ng/mL) for the PLMs [33].

### 4.2. Bactericidal Assay

Cell survival after treatment with different types of active and heat-inactivated sera was assessed. Sheep serum was obtained in previous intramammary infection experiments from 1.5 to 2 year-old lactating ewes of a local mountain breed, previously tested to be free of major sheep pathogens, including mycoplasmas, and seronegative for *M. agalactiae* (attested by routine bacteriological and PCR tests and *M. agalactiae*-specific commercial ELISA kits). The sheep were housed at the stables of the University of Veterinary Medicine Vienna in accordance with the guidelines of the University Ethic Commission and Austrian law for animal protection. The serum was obtained from the same sheep four days before inoculation (Day-4 p.i., D-4; non-sensitized serum) and 17 days after experimental infection (Day 17 p.i., D+17) with 10^9^ CFU of *M. agalactiae* type strain PG2 in PBS via the right teat canal as described earlier [12]. The latter serves as a sensitized serum as it contains *M. agalactiae*-specific antibodies due to seroconversion as shown earlier for this time point [12,15]. Other than sheep sera, guinea pig serum complement (Sigma-Aldrich, St. Louis, MO, USA) was used.

Heat inactivation of 1 mL sera aliquots was carried out at 56 °C in a water bath for 40 min for sheep sera, and 1 h for guinea pig serum. For the bactericidal assays, 50–100 µL aliquots of the mycoplasma culture were mixed with an equal amount of active or heat-inactivated serum, respectively. To keep a check on the equivalence of the starting bacterial counts, 5 µL were occasionally removed as controls at time point zero (T_0_). The mixture was then incubated for 1 h at 37 °C in a water bath (T_1_) as described previously for similar studies [16,17,22,24,34,35]. Standardization work concerning optimal incubation time and influence of suspension solution was performed during previous experiments [23].

The cell suspensions were serially diluted with SP-4 broth and aliquots were plated on SP-4 agar (1% Difco Agar Noble; BD, Franklin Lakes, NJ, USA). Due to the extremely small size of mycoplasma colonies, the optimal time-point for counting after incubation at 37 °C was determined in each case individually to ensure that no colony is missed even if smaller than the usual size. For instance, the PLMs take longer to grow to a visible size than the wild-type strains. After three to five days of incubation, the CFU were counted under a light stereomicroscope after flipping the agar plate in order for the agar side to be up and each counted colony marked with a pen. In case of an extensive number of densely populated colonies visible by the naked eye, 4 to 8 sectors were drawn on the agar plates and few of these were randomly counted instead of the whole plate to calculate the total CFU/plate.

### 4.3. Calculation of Percentage Survival

To calculate the serum resistance the CFU/mL for all cultures was determined. The CFU/mL for the active serum sample was then divided by CFU/mL for the heat-inactivated one to obtain the percentage survival as follows:

(1) $$\%\;\mathrm{survival}=\frac{\frac{\mathrm{CFU}}{\mathrm{mL}}\;\mathrm{of}\;\mathrm{sample}\;\mathrm{treated}\;\mathrm{with}\;\mathrm{active}\;\mathrm{complement}}{\frac{\mathrm{CFU}}{\mathrm{mL}}\;\mathrm{of}\;\mathrm{sample}\;\mathrm{treated}\;\mathrm{with}\;\mathrm{heat}-\mathrm{inactivated}\;\mathrm{complement}\;}\times 100$$

To assess whether there may have already been a difference in cell-count before the assay, which could in turn falsify the calculation of the killing ratio between the active and heat-inactivated complement, the T_0_ was also incorporated, especially at the beginning of the trials.

(2) $$\%\;\mathrm{survival}=\frac{\frac{\frac{\mathrm{CFU}}{\mathrm{mL}}{\;\mathrm{of}\;\mathrm{sample}\;\mathrm{treated}\;\mathrm{with}\;\mathrm{active}\;\mathrm{complement}\;\mathrm{of}\;T}_{1}}{\frac{\mathrm{CFU}}{\mathrm{mL}}{\;\mathrm{of}\;\mathrm{sample}\;\mathrm{treated}\;\mathrm{with}\;\mathrm{active}\;\mathrm{complement}\;\mathrm{of}\;T}_{0}}}{\frac{\frac{\mathrm{CFU}}{\mathrm{mL}}\;\mathrm{of}\;\mathrm{sample}\;\mathrm{treated}\;\mathrm{with}\;\mathrm{heat}-{\mathrm{inactivated}\;\mathrm{complement}\;\mathrm{of}\;T}_{1}}{\frac{\mathrm{CFU}}{\mathrm{mL}}\;\mathrm{of}\;\mathrm{sample}\;\mathrm{treated}\;\mathrm{with}\;\mathrm{heat}-{\mathrm{inactivated}\;\mathrm{complement}\;\mathrm{of}\;T}_{0}}\;}\times 100$$

### 4.4. Statistical Analysis

The resulting differences in serum resistance were analyzed in GraphPad Prism. Firstly, normality and the distribution of residuals were examined to evaluate the need for a parametric or non-parametric test. To see whether a significant change in CFU, and thereby a significant killing took place, the difference in CFU/mL of the active versus the heat-inactivated sample was assessed in an unpaired *t*-test or, for non-parametric distributions, in a Mann-Whitney test. Differences between the ratio of survival as calculated in 4.3 of PG2 and GM139 were further analyzed with an unpaired t-test or a Mann-Whitney test. The variability of percentage survival for the PLMs was analyzed by a two-way ANOVA with multiple comparisons. Results with a *p*-value of <0.05 were considered statistically significant.

### 4.5. Triton X-114 Phase Extraction & Blotting

Triton X-114 (Sigma Aldrich, St. Louis, MO, USA) phases of the GM139 and the PG2 strain were extracted as described elsewhere [36]. After 5 min at 95 °C under reducing conditions, samples were analysed by 10–12% sodium dodecylsulphate-polyacrylamide gel electrophoresis (SDS-PAGE). These were then blotted onto GE Healthcare Amersham^TM^ Protran^TM^ 0.2 µm NC (Thermo Fisher Scientific, Waltham, MA, USA) nitrocellulose membranes using blotting buffer (250 mM Tris, 1.92 M Glycine, 20% *v*/*v* Methanol) [6].

To test the reactivity of the D+17 sensitized sheep serum the nitrocellulose membrane was treated with sheep serum in a dilution of 1:100 in TBS and rabbit anti-sheep IgG, conjugated to horseradish peroxidase (DakoCytomation, Glostrup, Denmark) in a dilution of 1:2000 in TBS as secondary antibody.

## 5. Conclusions

Strains bearing different Vpma profiles exhibit altered serum and complement susceptibilities, with the PG2 strain being less resistant than the GM139 strain in all cases. PLMX, the mutant expressing the smallest Vpma, was the only one sensitive to guinea pig serum complement. Overall data of this study points towards higher susceptibility of the strains to sensitized serum, which in turn indicates that antibodies likely play a significant role in serum-killing of *M. agalactiae*. This is also emphasized by the fact that there was almost no bactericidal effect of the guinea pig serum complement which lacked *M. agalactiae*-specific antibodies. The PLMs with the smallest Vpmas (PLMU and PLMX) were the most susceptible compared to those expressing longer variants. Moreover, the increased fitness of PLMY in comparison to PLMU was once again proven, with PLMY being resistant to sensitized serum to a much greater extent.

## Data Availability

All relevant data are already present in this manuscript or in the Supplementary Materials.

## Supplementary Materials

The following supporting information can be downloaded at: https://www.mdpi.com/article/10.3390/pathogens11091036/s1, Figure S1: Comparative serum killing differences between *M. agalactiae* type strain PG2 and another field strain GM139 treated with *M. agalactiae*-sensitized sheep serum obtained on Day 17 post infection. Serum killing differs significantly between these two wild-type strains with **** *p* < 0.0001. Figure S2: Comparative serum killing differences between the six different Vpma Phase-Locked Mutants (PLMs) treated with *M. agalactiae*-sensitized sheep serum obtained on Day 17 post infection. Mean survival percentages are significantly different between all the Phase-Locked Mutants except for Phase-Locked Mutant U (PLMU) vs. Phase-Locked Mutant X (PLMX), Phase-Locked Mutant V (PLMV) vs. Phase-Locked Mutant Z (PLMZ), and Phase-Locked Mutant W (PLMW) vs. Phase-Locked Mutant Y (PLMY). *** *p* < 0.001; **** *p* < 0.0001. Figure S3: Comparative serum killing differences between *M. agalactiae* type strain PG2 and another field strain GM139 treated with guinea pig serum complement. Serum killing differs significantly between these two wild-type strains with **** *p* < 0.0001. Figure S4: Comparative serum killing differences between the six different Vpma Phase-Locked Mutants (PLMs) treated with guinea pig serum complement. Mean survival values are significantly different only between Phase-Locked Mutant X (PLMX) and the other five Phase-Locked Mutants. *** *p* < 0.001.
