# Supplementary material for: Serum Resistance of Mycoplasma agalactiae Strains and Mutants Bearing Different Lipoprotein Profiles
Source: Pathogens. 2022 Sep 13;11(9):1036. doi: 10.3390/pathogens11091036 (PMC9501237; doi:10.3390/pathogens11091036)
Supplement: Supplementary file 1 [file pathogens-11-01036-s001.zip › pathogens-1861439-supplementary.pdf]

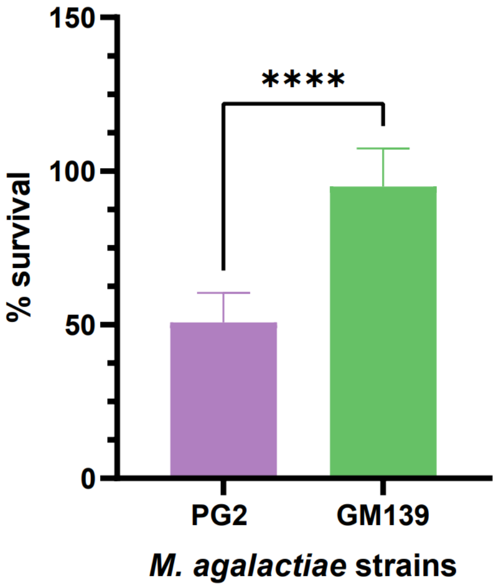


**Figure S1.** Comparative serum killing differences between *M. agalactiae* type strain PG2 and another field strain GM139 treated with *M. agalactiae*-sensitized sheep serum obtained on Day 17 post infection. Serum killing differs significantly between these two wild-type strains with **** $p < 0.0001$.

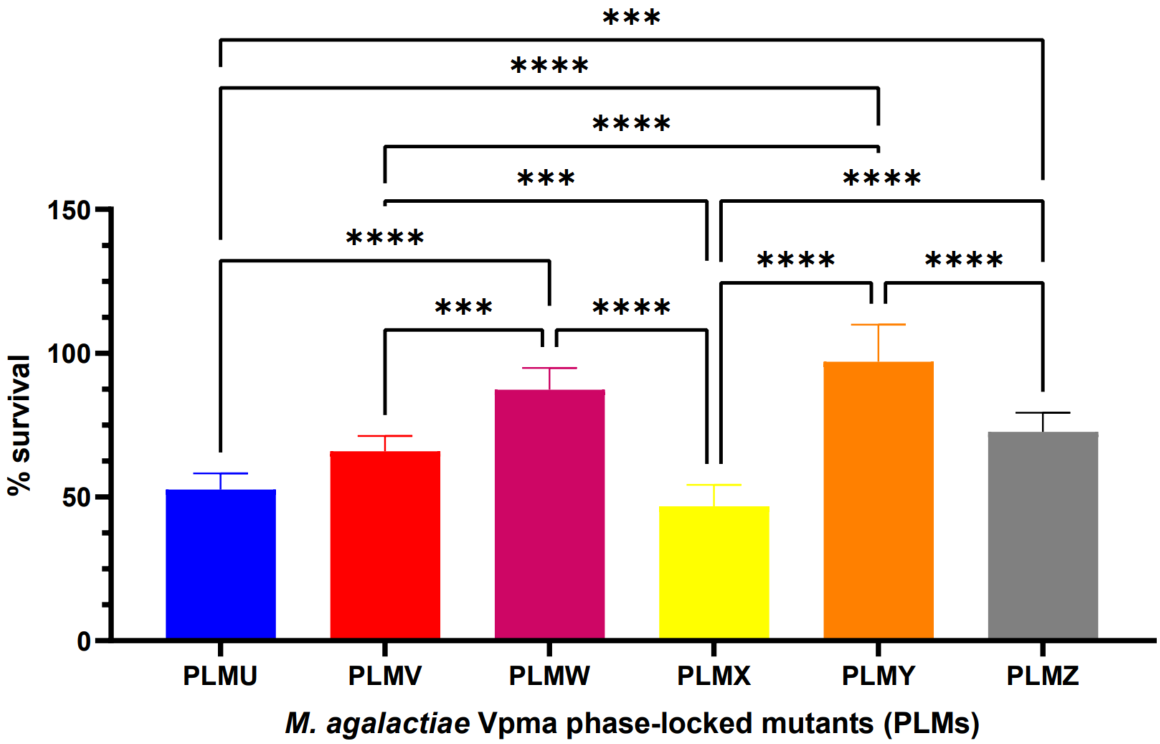


**Figure S2.** Comparative serum killing differences between the six different Vpma Phase-Locked Mutants (PLMs) treated with *M. agalactiae*-sensitized sheep serum obtained on Day 17 post infection. Mean survival percentages are significantly different between all the Phase-Locked Mutants except for Phase-Locked Mutant U (PLMU) vs. Phase-Locked Mutant X (PLMX), Phase-Locked Mutant V (PLMV) vs. Phase-Locked Mutant Z (PLMZ), and Phase-Locked Mutant W (PLMW) vs. Phase-Locked Mutant Y (PLMY). *** $p < 0.001$; **** $p < 0.0001$.

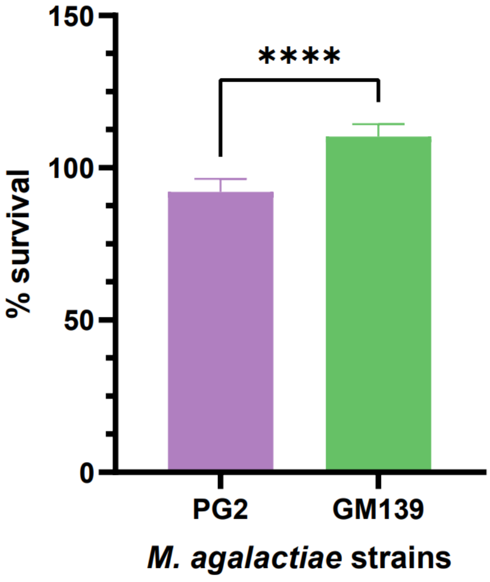


**Figure S3.** Comparative serum killing differences between *M. agalactiae* type strain PG2 and another field strain GM139 treated with guinea pig serum complement. Serum killing differs significantly between these two wild-type strains with **** $p < 0.0001$.

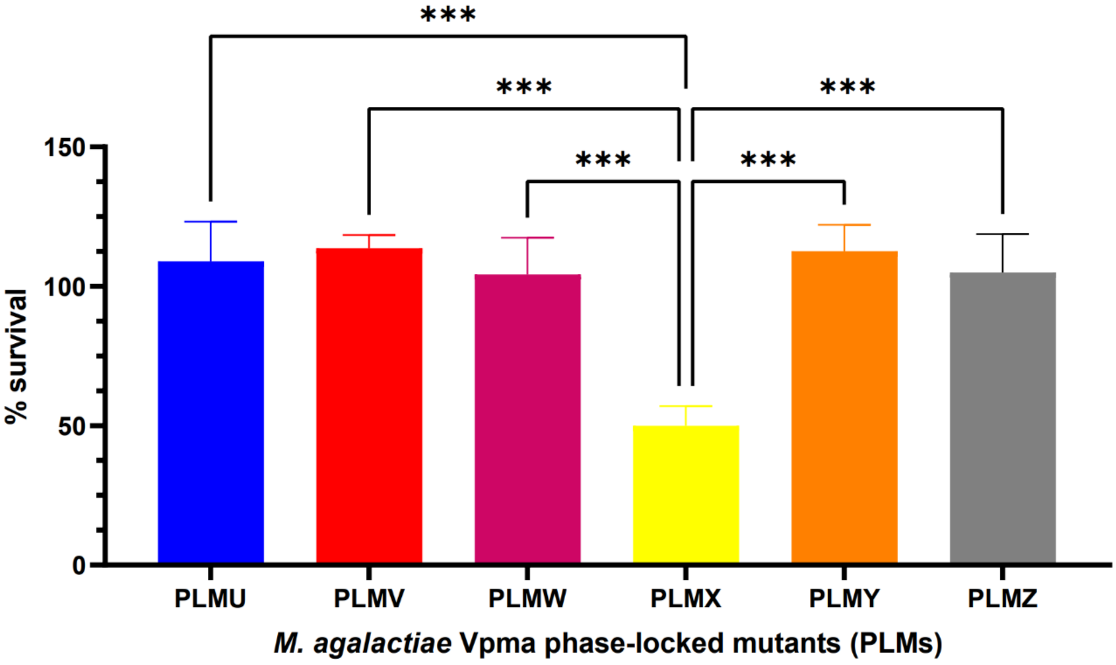


**Figure S4.** Comparative serum killing differences between the six different Vpma Phase-Locked Mutants (PLMs) treated with guinea pig serum complement. Mean survival values are significantly different only between Phase-Locked Mutant X (PLMX) and the other five PLMs. *** $p < 0.001$.
